# Threshold Models for Genome-Enabled Prediction of Ordinal Categorical Traits in Plant Breeding

**DOI:** 10.1534/g3.114.016188

**Published:** 2014-12-23

**Authors:** Osval A. Montesinos-López, Abelardo Montesinos-López, Paulino Pérez-Rodríguez, Gustavo de los Campos, Kent Eskridge, José Crossa

**Affiliations:** * Facultad de Telemática, Universidad de Colima, Avenida Universidad 333, Colima, México; † Departamento de Estadística, Centro de Investigación en Matemáticas (CIMAT), Guanajuato, Guanajuato, México; ‡ Colegio de Postgraduados, Edo. de México, México; § Department of Biostatistics, University of Alabama at Birmingham, 443, Birmingham, Alabama 35294; ** Department of Statistics, University of Nebraska, Lincoln, Nebraska; †† Biometrics and Statistics Unit, International Maize and Wheat Improvement Center (CIMMYT), México, D.F., México

**Keywords:** prediction accuracy, threshold model, disease resistance, GBLUP, genotype × environment interaction, GenPred, shared data resource

## Abstract

Categorical scores for disease susceptibility or resistance often are recorded in plant breeding. The aim of this study was to introduce genomic models for analyzing ordinal characters and to assess the predictive ability of genomic predictions for ordered categorical phenotypes using a threshold model counterpart of the Genomic Best Linear Unbiased Predictor (*i.e.*, TGBLUP). The threshold model was used to relate a hypothetical underlying scale to the outward categorical response. We present an empirical application where a total of nine models, five without interaction and four with genomic × environment interaction (G×E) and genomic additive × additive × environment interaction (G×G×E), were used. We assessed the proposed models using data consisting of 278 maize lines genotyped with 46,347 single-nucleotide polymorphisms and evaluated for disease resistance [with ordinal scores from 1 (no disease) to 5 (complete infection)] in three environments (Colombia, Zimbabwe, and Mexico). Models with G×E captured a sizeable proportion of the total variability, which indicates the importance of introducing interaction to improve prediction accuracy. Relative to models based on main effects only, the models that included G×E achieved 9–14% gains in prediction accuracy; adding additive × additive interactions did not increase prediction accuracy consistently across locations.

Since genomic selection (GS) initially was proposed by Meuwissen *et al.* (2001), a large number of plant breeding studies have assessed the prediction accuracy of GS in different economically important crops. These studies have used different marker platforms and marker densities, as well as different parametric and nonparametric statistical models (*e.g.*, de los Campos *et al.* 2009, 2010; Heffner *et al.* 2009, 2010; Crossa *et al.* 2010, 2011, 2013a,b; Heslot *et al.* 2012; Pérez-Rodríguez *et al.* 2010, 2012). Both simulation and empirical studies have shown that GS has greater prediction accuracy than standard pedigree-based prediction, and most of the benefits of GS arise from obtaining accurate predictions early in the breeding cycle. The success of genomic prediction is affected by the choice of model, the size of the training data, the heritability of the trait, the span of linkage disequilibrium, the marker density, and the strength of the genetic relationships between the training and validation populations, among other factors. A number of studies have assessed how these factors affect prediction accuracy for quantitative traits (Kizilkaya *et al.* 2014); however, little has been done with categorical traits. As first pointed out by Gianola (1980, 1982) and Gianola and Foulley (1983), these traits cannot be dealt with by the use of Gaussian models.

Several studies support the notion that when the number of categories is large and the data seem to follow an approximately normal distribution, failure to address the ordinality of the data is likely negligible (Atkinson 1988). However, significant bias is observed when the number of categories is less than five and sample size is not large enough. In addition, analyzing the data as normally distributed has less power of detection of effects and often produces estimates of frequency categories outside the 0−1 range, which does not make sense. These problems often occur when the frequencies are small or very large. Also, even when treatment means obtained with a normal approximation can be interpreted as estimates of the proportions of each category, this is not the case for the variance of their estimates as the data are binary and polychotomous (Stroup 2012). If the data are transformed, many of the aforementioned problems remain in a linear model analysis, given that the most commonly used transformations are intended to stabilize variance but fail to address the problem of skewness. Consequently, transformations often are ineffective and, in addition, express the data on scales that are unfamiliar to those who use the results of the analysis (Littell *et al.* 2002).

Categorical traits are scored by assigning a data point to one of several mutually exclusive and exhaustive ordered categories. If these scores are treated as continuous variables, as in standard GS linear models, the following assumptions do not hold: (1) the relationship between genomic data and phenotypes is linear; (2) phenotypes follow a normal distribution; and (3) the variance is constant and not a function of the expected value. Therefore, standard GS linear models currently used in plant and animal breeding do not meet the assumptions required for categorical data.

Results of empirical and simulation studies indicate that generalized linear mixed models (GLMMs) provide more sensible results and have greater power to identify model effects as statistically significant (Stroup 2012). In a linear model, the response variable (observation) equals the sum of explanatory variables plus a residual, and a probability assumption about the residual is made. In GLMM, the statistical model for a multinomial variable is expressed in a probability distribution form. It is important to point out that the cumulative probit model (also known as the threshold model) assumes that the process that gives rise to the observed categories is an underlying continuous variable with a standard normal distribution that in many applications uses the linear predictor with reversed signs for the fixed and random effects: $\eta_{c}=\gamma_{c}+\mathrm{X\beta}+\mathrm{Zb}$.

Threshold models have been used in animal GS to relate a hypothetical underlying scale to the outward categorical response. For example, González-Recio and Forni (2011) developed versions of BayesA, Bayesian Lasso, and two machine-learning methods for analyzing dichotomous traits and showed that the differences between methods are small, particularly with a large number of quantitative trait loci. On the other hand, Villanueva *et al.* (2011) developed a version of the BayesB method for dichotomous traits and concluded that the threshold BayesB method improves GS accuracy when disease-resistant dichotomous phenotypes are encountered, compared with accuracies obtained with the linear model. The threshold model showed an increase in accuracy of up to 16%, as well as significant advantages when heritability and disease prevalence were low and individuals were genotyped but not measured (testing set). Both González-Recio and Forni (2011) and Villanueva *et al.* (2011) pointed out that the models they developed for dichotomous phenotypes can easily be extended to traits with more than two discrete categories.

Recently, Wang *et al.* (2012) proposed threshold models using the GS framework and extended three Bayesian methods (BayesA, BayesB, and BayesCπ) to estimate genomic breeding values of animal threshold traits with more than two categories; they named these methods extended BayesTA, extended BayesTB, and extended BayesTCπ. They derived computing procedures for the three BayesT methods by using the Gibbs sampler algorithm. Through a simulation study, they found that the three BayesT methods generally performed better than the corresponding standard Bayesian methods, especially when only two phenotypic categories are present. They also found that BayesTB and BayesTCπ produced similar accuracies and that both performed better than BayesTA. Also, they addressed how heritability, number of quantitative trait loci, incidence, and number of phenotypic categories affect the performance of the three BayesT methods. Recently, Kizilkaya *et al.* (2014) proposed performing genomic prediction of ordinal categorical phenotypes using BayesTCπ and compared it with BayesCπ; they found that the accuracies of BayesTCπ for ordinal categorical phenotypes were greater than those of BayesCπ and that there was a greater advantage in using BayesTCπ when the training population was small. These authors pointed out that a 2.25-fold increase in the training population size for ordinal categorical phenotypes analyzed as a linear model using BayesCπ was sufficient to achieve an accuracy equal to or greater than that for continuous phenotypes with a training population size of 1000 individuals.

In plant breeding, the most economically important trait is grain yield, which is greatly affected by environmental factors, as well as by genotype × environment interaction (G×E). The GS models first introduced by Meuwissen *et al.* (2001) were further extended into multienvironment models that can handle large numbers of individuals genotyped with large numbers of markers and evaluated in multiple environments. Burgueño *et al.* (2012) analyzed multienvironment data using a multivariate version of the genomic best linear unbiased predictor (GBLUP). Jarquín *et al.* (2014) extended GBLUP to incorporate highly dimensional markers, environmental covariates, and their interactions into a class of random-effects models where main and interaction effects are modeled using Gaussian processes with covariance functions based on genetic and environmental similarity among entries. In their study, Jarquín *et al.* (2014) used as a covariance function the structure induced by a reaction norm model, and applied the proposed approach to the grain yield trait in wheat lines evaluated over multiple years and locations.

However, in plant breeding, many economically important traits, such as the degree of resistance/susceptibility, are categorical. Despite this, to our knowledge, threshold models have not been considered in GS for plant breeding. Therefore, in this study, we introduced a threshold GS model that is an extension of the GBLUP of Jarquín *et al.* (2014) which incorporates G×E and additive × additive × environment (G×G×E) interactions. The proposed model was evaluated using a data set consisting of 278 maize lines scored for resistance to gray leaf spot (GLS) measured using an ordered categorical scale [1 (no disease) to 5 (complete infection)] in three environments and genetic information consisting of 46,347 single-nucleotide polymorphisms (SNPs).

## Materials and Methods

### Experimental data

#### Phenotypic data:

The trait analyzed in 278 maize lines was gray leaf spot (GLS), caused by the fungus *Cercospora zeae-maydis*, which was evaluated in three environments, Mexico, Zimbabwe, and Colombia. GLS is one of the most important foliar diseases of maize worldwide. The GLS trait was analyzed using an ordinal scale from 1 (no disease), 2 (low infection), 3 (moderate infection), 4 (high infection), to 5 (complete infection). These data are part of the data set previously analyzed by Crossa *et al.* (2011) and González-Camacho *et al.* (2012), among others.

#### Genotypic data:

Genotypes of all 278 lines were obtained using 53,401 SNPs. Those SNPs with >10% missing values or a minor allele frequency ≤0.05 were excluded from the data. Call rate per line was considered sufficient (>90%) for all lines. Most lines (250 of 278) had a call rate greater than 99%. After line-specific quality control (applying the same quality control to each line separately), the maize data still contained 46,347 SNPs, which were used in the analysis.

### Statistical models

#### Threshold (cumulative probit) model:

Let $y=\{y_{ijk}\}$ ($i=1,\ldots ,I;j=1,2,\ldots ,J;k=1,2,\ldots r_{ij},$ where i represents the environment, *j* denotes the genotype and *k* is the number of replicates of each genotype in each environment. The response variable (disease resistance), $y_{ijk}$, represents an assignment into one of C mutually exclusive and exhaustive categories (here C=5, I = 3 and J = 278) that follow an order, since 1 indicates no infection, 2 low infection, 3 moderate infection, 4 high infection, and 5 complete infection. Therefore, in a GLMM framework, this model can be described by defining the distribution, the linear predictor and the link function.

##### Distribution:

There are two distributions, one for observations in the response variable: $\left(y_{1ij},y_{2ij},\ldots ,y_{Cij}|\beta ,b\right)∼\mathrm{Multinomial}\left(N_{ij},\pi_{1ij},\pi_{2ij},\ldots ,\pi_{Cij}\right)$, where β is the I×1 vector of fixed environmental effects and another distribution for the random effects, b∼N(0,G), where **G** is the variance-covariance matrix of $b=\left\{b_{j}\right\}$ and $b_{j}$ is the effect of line j.

##### Linear predictor:

$\eta_{cij}=\gamma_{c}-x_{ij}^{T}\beta -z_{ij}^{T}b$, where $\eta_{cij}$ denotes the c^th^ link (c=1,2,…,C−1) for the fixed and random effects combination, $\gamma_{c}$ is the intercept (threshold) for the c^th^ link, and $x_{ij}^{T}$ and $z_{ij}^{T}$ are known row incidence vectors corresponding to fixed and random effects in β and b, respectively. Since there are C categories, a total of C−1 link functions are required to fully specify the model.

##### Link function:

Cumulative probit $\left\{\eta_{1ij}={\text{Φ}}^{-1}\left(\pi_{1ij}\right),\eta_{2ij}={\text{Φ}}^{-1}\left(\pi_{1ij}+\pi_{2ij}\right),\ldots ,\eta_{(C-1)ij}={\text{Φ}}^{-1}(\pi_{1ij}+\pi_{2ij}+\ldots +\pi_{\left(C-1\right)ij})\right\}$.Φ(⋅) is the cumulative distribution function of a standard normal distribution (probit link) and ${\text{Φ}}^{-1}$ its corresponding inverse.

The inverse link for this model is given as follows: $\pi_{1ij}=\text{Φ}\left(\eta_{1ij}\right),\pi_{1ij}+\pi_{2ij}=\text{Φ}\left(\eta_{2ij}\right),\ldots ,\pi_{1ij}+\pi_{2ij}+\ldots +\pi_{\left(C-1\right)ij}=\text{Φ}\left(\eta_{\left(C-1\right)ij}\right)$. Once we have estimates of $\text{Φ}\left(\eta_{1ij}\right),\text{Φ}\left(\eta_{2ij}\right),\ldots ,\text{Φ}\left(\eta_{\left(C-1\right)ij}\right),$ we estimate ${\hat{\pi }}_{2ij}=\text{Φ}\left(\eta_{2ij}\right)-\text{Φ}\left(\eta_{1ij}\right)$, ${\hat{\pi }}_{3ij}=\text{Φ}\left(\eta_{3ij}\right)-\text{Φ}\left(\eta_{2ij}\right)$,…,${\hat{\pi }}_{Cij}=1-\text{Φ}\left(\eta_{(C-1)ij}\right)$. This threshold model assumes that the process that gives rise to the observed categories is an underlying continuous variable with a normal distribution $l_{ijk}=x_{ij}^{T}\beta +z_{ij}^{T}b+\varepsilon_{ijk},$ where $l_{ijk}$ are called “liabilities,” $\varepsilon_{ijk}∼N\left(0,1\right)$ (*e.g.*, Gianola 1982, and Sorensen *et al.* 1995). Furthermore, to generate ordinal categorical phenotypes with C categories, the underlying phenotypic values are mapped to ordinal categorical phenotypes based on threshold parameters $\gamma^{T}=\left(\gamma_{min}<\gamma_{1}<\cdots <\gamma_{C-1}<\gamma_{max}\right)$ with $\gamma_{min}=-\infty$, and $\gamma_{max}=\infty ,$ which are *cutpoints* of the continuous scale such that the observed ordinal categorical response ($y_{ijk})$ is given by:

$$y_{ijk}=\{\begin{array}{c} 1\;if-\infty <l_{ijk}<\gamma_{1},\\ 2\;if\;\gamma_{1}<l_{ijk}<\gamma_{2},\\ \vdots \\ C\;if\;\gamma_{C-1}<l_{ijk}<\infty \end{array}$$

That is, $y_{ijk}$ falls into category C when the latent variable falls into the *c*^th^ interval of values. Given β and b, $\text{the liabilities }l_{ijk}$ are conditionally independent and distributed as

$$l_{ijk}|\beta ,b∼N(x_{ij}^{T}\beta +z_{ij}^{T}b,\sigma_{\varepsilon }^{2}=1)$$

where $\sigma_{\varepsilon }^{2}$ is fixed to 1 to achieve identifiability in the likelihood because the liabilities are unobservable. Also, one of the thresholds was fixed ($\gamma_{1}=0)$ to center the distribution. Therefore,

$$P\left(l_{ijk}\leq \gamma_{c}|\beta ,b\right)=\Phi \left(\gamma_{c}-x_{ij}^{T}\beta -z_{ij}^{T}b\right)\;\mathrm{for}\;c=1,2,\ldots ,C-1.$$

Note that $\gamma_{c}-x_{ij}^{T}\beta -z_{ij}^{T}b$ is the predictor of the GLMM for the multinomial data given previously. Then, the conditional probability that $y_{ijk}$ falls into category c (c=1,…,5) given β,b, and $\gamma =(\gamma_{min},\gamma_{1},\gamma_{2},\gamma_{C-1},\gamma_{max}),$ is given by

$$\begin{array}{cc} P(y_{ijk}=c|\beta ,b,\gamma ) & =P(\gamma_{c-1}<l_{ijk}<\gamma_{c}|\beta ,b,\gamma )\\ & =\text{Φ}\left(\gamma_{c}-x_{ij}^{T}\beta -z_{ijk}^{T}b\right)-\text{Φ}\left(\gamma_{c-1}-x_{ij}^{T}\beta -z_{ij}^{T}b\right)(1) \end{array}$$

The data are assumed conditionally independent, given β, b, and γ. Therefore, the sampling model is

$$\begin{array}{ll} p(y|\beta ,b,\gamma ) & =\overset{I}{\underset{i=1}{\prod }}\overset{J}{\underset{j=1}{\prod }}\overset{r_{ij}}{\underset{k=1}{\prod }}\overset{C}{\underset{c=1}{\sum }}I_{\left\{y_{ijk}=c\right\}}P(y_{ijk}|\beta ,b,\gamma )\\ & =\overset{I}{\underset{i=1}{\prod }}\overset{J}{\underset{j=1}{\prod }}\overset{r_{ij}}{\underset{k=1}{\prod }}\overset{C}{\underset{c=1}{\sum }}I_{\left\{y_{ijk}=c\right\}}[\text{Φ}\left(\gamma_{c}-x_{ij}^{T}\beta -z_{ij}^{T}b\right)-\text{Φ}\left(\gamma_{c-1}-x_{ij}^{T}\beta -z_{ij}^{T}b\right)] \end{array}$$

where $I_{\left\{y_{ijk}=c\right\}}$ is an indicator function taking a value of 1 if the response falls into category c, and 0 otherwise.

#### Models fitted:

Based on Jarquín *et al.* (2014), nine models are proposed for analyzing these data sets (Table 1). The sequence of models described below is similar to those presented by Jarquín *et al.* (2014) for a continuous variable. However, for simplicity, only the underlying latent variables ($l_{ijk})$ are presented together with the distribution of the corresponding random effects that give rise to the observed categorical phenotypes, given that a probit link function is assumed by all models described below.

**Table 1:** Nine models used to fit the data set

Model	Main Effects			Interaction		
	E	L	G	G×G	G×E	G×G×E
1	X	X				
2	X		X			
3	X		X	X		
4	X	X	X			
5	X	X	X	X		
6	X		X		X	
7	X		X	X	X	X
8	X	X	X		X	
9	X	X	X	X	X	X

E, environment; L, line; G, marker covariates; G×G, additive × additive epistasis term; G×E, environment × marker interaction; G×G×E, additive × additive epistasis × environment interaction term.

##### Model 1:

The first model used to explain the liability value of the *k*^th^ individual in the *j*^th^ line at the *i*^th^ environment is

$$l_{ijk}=E_{i}+L_{j}+\varepsilon_{ijk}$$ (2)

where $E_{i}$ is the fixed effect of the *i*^th^ environment and coefficient regressions were assigned a flat Gaussian prior with mean zero and variance equal to $1\times 10^{10}$, $L_{j}$ is the random effect of the *j*^th^ line which is assumed to be identically and independently distributed (*IID*) as normal, $L_{j}\overset{IID}{∼}N\left(0,\sigma_{L}^{2}\right),$ and $\varepsilon_{ijk}$ is an error term distributed as $\varepsilon_{ijk}\overset{IID}{∼}N(0,1)$. The unknown variance parameters ($\sigma_{L}^{2})$ were assigned scaled-inverted χ^2^ distributions as prior. This density is indexed by two hyper-parameters: degrees of freedom (*df*) and the scale (*S*) parameter. In this case, we defined these values using the internal rules provided by BGLR. By default, that is, if the user does not specify these parameters, BGLR assigns mildly informative priors with df=5 and a scale parameter that gives a prior mode, $\frac{S}{df+2}$, that obeys a prior variance partition where 50% of the variance of the liability score corresponds to error terms, and the remaining 50% to the random effects included in the model. The internal rules implemented in BGLR are fully explained in Pérez-Rodríguez and de los Campos (2014).

##### Model 2:

The second model is an extension of the GBLUP for ordered categorical phenotypes. This model was obtained from model 1 by replacing the line effect, $L_{j}$, with a random effect that incorporates marker information using the genomic relationship matrix ***G***. Model 2 expresses the liability value of the *k*^th^ individual in the *j*^th^ line in the *i*^th^ environment as

$$l_{ijk}=E_{i}+g_{j}+\varepsilon_{ijk}$$ (3)

with $g_{j}=\overset{p}{\underset{m=1}{\sum }}x_{jm}b_{m},$ where $g_{j}$ represents an approximation to the true genetic value of the *j*^th^ line, $x_{jm}$ is the genotype of the *j*^th^ line at the *m*^th^ marker (scored as 0 or 2 for genotypes that are homozygous at minor and major allele frequency, respectively, and 1 for heterozygous genotypes), and $b_{m}$ is the effect of the *m*^th^ marker with $b_{m}\overset{IID}{∼}N(0,\sigma_{b}^{2})$, (m=1,…,p). Therefore, $g=Xb_{m}$ contains the genomic values of all lines, and $g=\left(g_{1},\ldots ,g_{278}\right)\prime$ is assumed to follow the normal distribution $g∼N\left(0,G\sigma_{g}^{2}\right),$ where G is a marker-derived genomic relationship matrix that has an expected value equal to (under ideal conditions) twice the coefficient of parentage matrix of lines, and $\sigma_{g}^{2}$ is a genomic variance.

The entries of G were computed as $G_{jj^{’}}=p^{-1}\overset{p}{\underset{m=1}{\sum }}z_{jm}(z_{j^{’}m})$, where $z_{jm}=(x_{jm}-2p_{m})/\sqrt{2p_{m}(1-p_{m})}$ and $p_{m}$ is the estimated frequency of the *m*^th^ marker. Subtracting $2p_{m}$ from the genotype codes (centering) and dividing each marker covariate by $\sqrt{2p_{m}(1-p_{m})}$ (standardizing) is not strictly needed; however, standardization allows interpreting $\sigma_{g}^{2}$ as a genomic variance. G was constructed using the genotypes of all 278 lines.

##### Model 3:

The third model is an extension of model 2 that accounts for “marked” epistatic additive × additive relationships, $g_{A}∼N\left(0,G_{A}\sigma_{Ag}^{2}\right),$ where $g_{A}$ represents a regression on genomic epistatic additive × additive relationships with $G_{A}=G\#G$ (# is the element-wise multiplication operator, that is, a Hadamard product). The prior used for $\sigma_{Ag}^{2}$ was a scaled-inverted χ^2^ distribution. In this model, the liability score of the *k*^th^ individual in the *j*^th^ line in the *i*^th^ environment is equal to

$$l_{ijk}=E_{i}+g_{j}+g_{Aj}+\varepsilon_{ijk}$$ (4)

##### Model 4:

The third model combines model 1 and model 2 as

$$l_{ijk}=E_{i}+L_{j}+g_{j}+\varepsilon_{ijk}$$ (5)

Model 4 partitions the line effects into two components, one that is explained by a regression on markers ($g_{j}$) and another representing variation among lines that is not explained by regression on markers.

##### Model 5:

This model is an extension of model 4 that accounts for epistatic additive × additive relationships, with liability equal to

$$l_{ijk}=E_{i}+L_{j}+g_{j}+g_{Aj}+\varepsilon_{ijk}$$ (6)

Note that none of the aforementioned models accounts for G×E ($gE_{ij})$ and G×G×E ($g_{A}E_{ij})$ interactions. Next, we consider models that incorporate G×E and G×G×E.

##### Model 6:

Model 6 considers model 2 but, in addition to the main effects of environments (E) and markers (G), it takes into account the interaction between markers and environments (G×E). Because the data include multiple phenotypic records per line, the genetic covariance structure of genetic effects in the full data set is equal to $Z_{g}GZ_{g}^{T}$, where $Z_{g}$ is the incidence matrix for the vector of additive genetic effects of markers (Jarquín *et al.* 2014). Therefore, the covariance structure for the vector of interaction terms in the full data set $\mathrm{gE}=\left\{gE_{ij}\right\}$ is the Hadamard product of $Z_{g}GZ_{g}^{T}$ and $Z_{E}Z_{E}^{T},$ where $Z_{E}$ represents the incidence matrix of the effects of environments (*i.e.*, the matrix that connects phenotypes with environments) (Jarquín *et al.* 2014). Therefore, the liability value of the *k*^th^ individual in the *j*^th^ line in the *i*^th^ environment is explained by

$$l_{ijk}=E_{i}+g_{j}+gE_{ij}+\varepsilon_{ijk}$$ (7)

where $g∼N\left(0,G\sigma_{g}^{2}\right),\mathrm{gE}∼N(0,Z_{g}GZ_{g}^{T}\#Z_{E}Z_{E}^{T}\sigma_{gE}^{2})$ and $\varepsilon_{ijk}\overset{IID}{∼}N\left(0,1\right)$.

##### Model 7:

Model 7 extends model 6 to account for marked epistatic additive × additive relationships and the interaction between the epistatic additive × additive term and the environments. The liability value of the *k*^th^ individual in the *j*^th^ line in the *i*^th^ environment is now

$$l_{ijk}=E_{i}+g_{j}+g_{Aj}+gE_{ij}+g_{A}E_{ij}+\varepsilon_{ijk}$$ (8)

where $g_{A}E∼N(0,Z_{g}G_{A}Z_{g}^{T}\#Z_{E}Z_{E}^{T}\sigma_{g_{A}E}^{2})$ with $G_{A}=G\#G$.

##### Model 8:

Model 8 extends model 6 by adding the random effect of the lines ($L_{j}$). Thus, the liability value of the *k*^th^ individual in the *j*^th^ line in the *i*^th^ environment is explained by

$$l_{ijk}=E_{i}+L_{j}+g_{j}+gE_{ij}+\varepsilon_{ijk}$$ (9)

##### Model 9:

Finally, model 9 extends model 8 by adding marked epistatic additive × additive relationships and the interaction between the epistatic additive × additive term and the environments. The liability value of the *k*^th^ individual in the *j*^th^ line in the *i*^th^ environment is explained by 

$$l_{ijk}=E_{i}+L_{j}+g_{j}+g_{Aj}+gE_{ij}+g_{A}E_{ij}+\varepsilon_{ijk}$$ (10)

#### Implementation with BGLR:

The nine models were fitted using the R-package BGLR (de los Campos and Pérez-Rodríguez 2013) in the R-software (R Core Team 2014). Parametric and semi-parametric models with both molecular marker and pedigree data can be fitted in this R package. It also allows including various random effects with user-defined covariance matrices. In Appendix 1, we provide the R code used for fitting the most parameterized model (model 9). The implementation of the other models is similar to that of model 9 but with fewer random effects. All these models are implemented via a Bayesian approach using the Gibbs sampler algorithm, and sampling from the fully conditional distributions, as shown in Sorensen *et al.* (1995). For more details about Gibbs sampler implementation used in the R-package BGLR (de los Campos and Pérez-Rodríguez 2013) for binary and ordered categorical phenotypes, refer to Sorensen *et al.* (1995). The prior distributions of the variance components of all the models are described in the appendix of the BGLR package (de los Campos and Pérez-Rodríguez 2013).

#### Assessing predictive ability for ordered categorical phenotypic data:

The performance of the models was evaluated employing a cross-validation approach to estimate their prediction accuracies. To that end, we split each of the data sets into two parts (training and validation sets), where the training set was used to fit the model and the validation set was used to evaluate the training model’s prediction ability. A total of 20 random partitions were performed, and the validation results were averaged over the 20 partitions with observations assigned to training and testing completely at random. This represents a prediction problem similar to that labeled as CV2 in Burgueño *et al.* (2012), where the performance of some lines was observed in some environments (training set) but not in others (testing set).

Measuring prediction accuracy for categorical traits is more challenging than for quantitative traits, where the Euclidean metric appears as a natural choice. A variety of scoring rules have been proposed to assess accuracy for categorical traits. A scoring rule provides a summary measure for evaluating probabilistic prediction by assigning a numerical score based on the predictive distribution and on the event or value that materializes (Garthwaite *et al.* 2005; Kneib *et al.* 2007). This goal is achieved in a reasonable way by taking into account not only point prediction but the whole predictive distribution.

In the case of a multinomial model, this predictive distribution is simply obtained by computing the probabilities for all C categories of the response according to the estimated model, *i.e.*, we obtain the predictive distribution ${\hat{\pi }}_{i}=({\hat{\pi }}_{i1},{\hat{\pi }}_{i2},\ldots ,{\hat{\pi }}_{iC}$), derived from the estimated model for observation *i*, and $c_{i}$ is the realized value for this observation in the data set. A scoring rule is any real-value function $S({\hat{\pi }}_{ic},c_{i})$, $c_{i}=1,2,\ldots ,C$, that assigns a value to the event that category c is observed when ${\hat{\pi }}_{ic}$ is the predictive probability for individual i in category c. A suitable score is the sum

$$S=\overset{n}{\underset{i=1}{\sum }}S({\hat{\pi }}_{ic},c_{i})$$

where S is the sum over all observations in the test data set. The hit rate (*i.e.*, the percentage of true positive predictions) and the log-likelihood are two popular scoring rules. However, both have the drawback that they involve only one of the probabilities of the predictive distribution (${\hat{\pi }}_{i}$). In addition, it has been well documented that the log-likelihood score is sensitive to extreme observations. In our applications, we utilize the Brier score (Brier 1950), which is equal to

$$BS=n^{-1}\overset{n}{\underset{i=1}{\sum }}\overset{C}{\underset{c=1}{\sum }}{\left({\hat{\pi }}_{ic}-d_{ic}\right)}^{2}$$ (11)

where $d_{ic}$ takes the value of 1 if the ordinal categorical response observed for individual i falls into category c, and $d_{ic}=0\text{ otherwise}$. This scoring rule uses all the information contained in the predictive distribution, not just a small part such as the hit rate or the log-likelihood score. Therefore, it is a reasonable choice for comparing categorical regression models, even though there are other scoring rules that also have good properties. The range of BS in equation (11) is between 0 and 2. For this reason, we took BS/2, to get the Brier score bound between 0 and 1, and lower scores imply better predictions.

#### Data and software:

The phenotypic data for GLS in three environments (Mexico, Zimbabwe, and Colombia) for the 278 maize lines, the 46,347 SNPs data and the R scripts developed to fit the predictive models used in this study are given in the GLScode.rar file deposited at http://repository.cimmyt.org/xmlui/handle/10883/4128. The BGLR package (de los Campos and Pérez-Rodríguez 2013) can be downloaded from CRAN.

## Results

**Figure 1:**
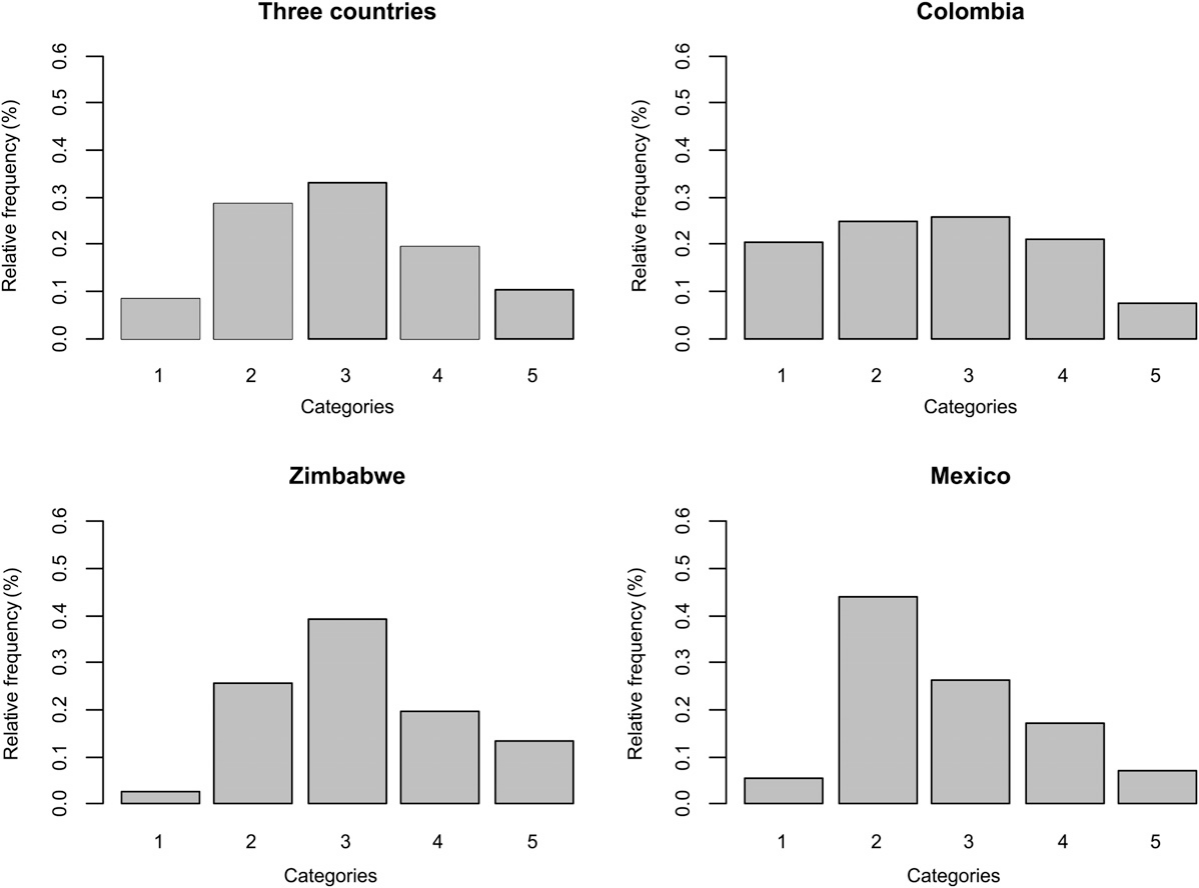
Relative frequency of each category in the whole data set.

Figure 1 shows the relative frequencies of each category for the whole data set and for each country. The whole data set contains 2798 observations; category 3 has the most observations (923), and category 1 is the one with the fewest (234). This pattern was also observed in Zimbabwe (1485 total observations; 37 in category 1 and 581 in category 3) but somewhat more pronounced. Colombia had 832 observations, with 215 in category 3, 208 in category 2, and only 63 in category 5. Mexico had 481 observations, most of them in category 2 (212), and category 1 had the fewest data (26 observations).

When applying eigenvalue decomposition to the genomic relationship matrix, 82 eigenvectors (components) of a total of 278 were needed to explain 80% of the total variance of genotypes. The first eigenvector captured 13.68% of the total variation of marker genotypes, whereas the second eigenvector explained 6.28% of the total variation. For these reasons, we can say that there is some, but not strong, evidence of population (and family) stratification, indicating a relatively diverse set of lines. This was expected because these lines came from different breeding programs.

**Figure 2:**
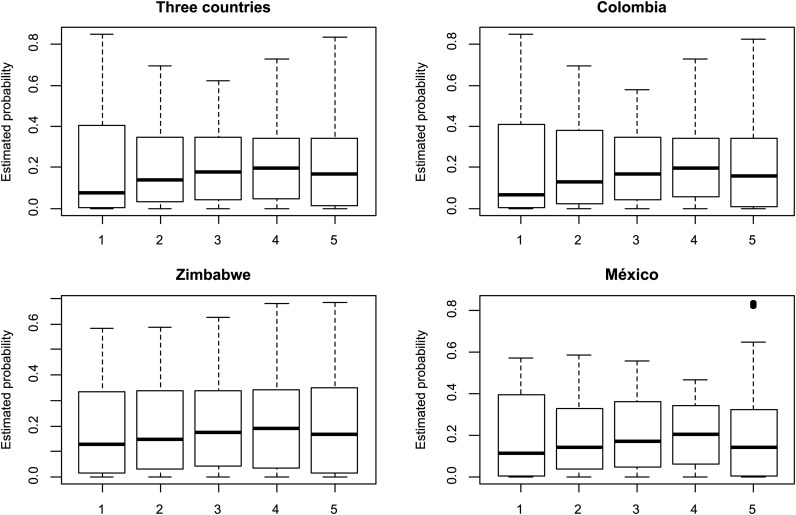
Estimated probability of each category in the whole data set and of each location in model 9.

Table 2 gives the estimates of the fixed (environment) effects and threshold parameters for each of the nine models; the first five models (1−5) had similar parameter estimates (fixed and threshold) that were different from those of models 6−9. Models 6−9 produced similar fixed and threshold parameter estimates. In plant and animal breeding, the focus is on estimating response probabilities associated with specific linear parameter combinations (Gianola and Foulley 1983). Figure 2 gives the probabilities for each ordinal categorical phenotype for model 9, for the whole data set, and for each country. In Figure 2, the average probabilities for category 5 (complete infection) were slightly greater than 0.20 (20%) in the whole data set and in each country. Colombia shows the greatest probability of complete infection (category 5), but it was only slightly greater than the probabilities in Mexico and Zimbabwe.

**Table 2:** Mean and SD of posterior distributions of fixed (environment) effects $\left(\beta_{1}\text{ for Colombia},\beta_{2}\text{ for Zimbawe},\text{and }\beta_{3}\text{ for Mexico}\right)$ and threshold parameters of the nine proposed models

Model	Mean Fixed Parameters			Mean Threshold Parameters			
	$\beta_{1}$	$\beta_{2}$	$\beta_{3}$	$\gamma_{2}$	$\gamma_{3}$	$\gamma_{4}$	$\gamma_{5}$
1	−2.5108	−1.9852	−2.3863	−3.7668	−2.5652	−1.6167	−0.8285
2	−2.5522	−2.0165	−2.4118	−3.8006	−2.6059	−1.6544	−0.8544
3	−2.5129	−2.0008	−2.3791	−3.7776	−2.5840	−1.6313	−0.8338
4	−2.5397	−2.0142	−2.4125	−3.7951	−2.5989	−1.6518	−0.8497
5	−2.5202	−1.9898	−2.3855	−3.7718	−2.5675	−1.6242	−0.8322
6	−3.4488	−2.7384	−3.2335	−5.1665	−3.4851	−2.2005	−1.1765
7	−3.4497	−2.7242	−3.2277	−5.1629	−3.4768	−2.2021	−1.1821
8	−3.4587	−2.7354	−3.2449	−5.1674	−3.4795	−2.2056	−1.1766
9	−3.4402	−2.7111	−3.2167	−5.1345	−3.4641	−2.1834	−1.1645
	SD Fixed Parameters			SD Threshold Parameters			
1	0.3362	0.3242	0.3381	0.3525	0.3479	0.3310	0.2951
2	0.3403	0.3284	0.3415	0.3566	0.3518	0.3366	0.3028
3	0.3422	0.3311	0.3438	0.3593	0.3545	0.3391	0.3031
4	0.3242	0.3132	0.3256	0.3389	0.3341	0.3215	0.2903
5	0.3321	0.3210	0.3338	0.3479	0.3429	0.3289	0.2956
6	0.4749	0.4515	0.4720	0.5324	0.4923	0.4490	0.3910
7	0.4801	0.4569	0.4770	0.5338	0.4953	0.4564	0.3997
8	0.4791	0.4571	0.4756	0.5316	0.4949	0.4561	0.4014
9	0.4819	0.4588	0.4783	0.5352	0.4981	0.4576	0.4007

SD, standard deviation.

It is important to point out that the average probabilities of high infection (category 4) were very similar to the average probabilities of complete infection (category 5) for the whole data set and for each country, and that no infection (category 1) was the category with the lowest average probabilities (around 15%). These probability estimates were very different from estimates obtained based on raw frequencies (Figure 1) because they take into account the distribution of records across countries, the structure of lines, and the interaction between markers and environments (countries). The linear predictor used for doing this calculation was taken from model 9, which is given in Equation (10).

Estimates of variance components derived from the full data analysis are given in Table 3. The interaction (G×E) explained the largest proportion of the variance of liabilities, with estimated posterior means greater than 1.03 (models 6−9). The total variance explained by model 1 was 1.20, which is 1.7659 times smaller than the total variance captured by the most parameterized model (model 9), with a total variance of 2.1191. Also, model 2 (with E and G as main effects) only captured a total variance equal to 1.1911, which was 1.7791 times smaller than the total variance captured by model 9. The variance explained by models 3, 4, and 5 were, respectively, 1.7755, 1.7767, and 1.782 times smaller than the variance captured by model 9. However, models 6, 7, and 8 captured a total variance equal to 2.1321, 2.0967, and 2.116, respectively, which were almost identical to the total variance captured by model 9; model 6 captured the highest variability. It should be noted that in models 6, 7, 8, and 9, the percentages of variance explained by the interaction term (G×E) were 51.68, 49.42, 51.23, and 50.19%, respectively, clearly indicating that the interaction term was the most important source of variability in these models. It should be noted that if interactions were omitted, a large proportion of the variability in models 6 and 8 was not captured by main effects and the variance of the error term $\varepsilon_{ijk}$ was the greatest source of variability. Another important finding was that the additive × additive epistatic terms explained 1.72, 1.06, 0.58, and 0.46% of the total variability in models 3, 5, 7, and 9, respectively, which means that adding this type of epistasis is not very important for this trait in these populations.

**Table 3:** Estimated variance components of the nine proposed models

Model	L	G	G×G	G×E	G×G×E	TotVar
1	0.2000 (16.67)					1.2000
2		0.1911 (16.04)				1.1911
3		0.1730 (14.50)	0.0205 (1.72)			1.1935
4	0.0112 (0.94)	0.1815 (15.22)				1.1927
5	0.0090 (0.76)	0.1676 (14.09)	0.0126 (1.06)			1.1892
6		0.0303 (1.42)		1.1018 (51.68)		2.1321
7		0.0165 (0.79)	0.0121 (0.58)	1.0362 (49.42)	0.0319 (1.52)	2.0967
8	0.0117 (0.55)	0.0202 (0.95)		1.0841 (51.23)		2.116
9	0.0080 (0.38)	0.0122 (0.58)	0.0097 (0.46)	1.0636 (50.19)	0.0256 (1.21)	2.1191

Numbers in parenthesis are the percentages of variance explained by each component. L, line; G, marker covariates; G×G, additive × additive epistasis term; G×E, environment × marker interaction; G×G×E, additive × additive epistasis × environment interaction term; TotVar, total variance explained by each model including the variance of $\varepsilon_{ijk},$ which is equal to 1.

Table 4 presents the Brier scores of the validation samples for each country and for the nine models. Because phenotype was ordinal categorical, we used Brier scores instead of Pearson’s correlation coefficient for assessing prediction accuracy, because the Brier scores are bounded between 0 and 1, and values closer to zero imply better prediction accuracy. In Table 4, models 6, 7, 8, and 9 presented the lowest Brier scores, so these four models had better prediction ability. Models 1−5 showed the worst prediction ability. In Colombia, the prediction ability of model 9 was 19.85% greater than that of model 1. In Mexico, the increase in the prediction ability of model 9 over model 1 (the simplest model) was 11.26%, while in Zimbabwe this increase was 8.71%. Although Table 3 showed that inclusion of the interaction term (G×E) explained the largest proportion of variability in models 6 and 8, this did not produce a large increase in the prediction ability of these two models over that of the models without interactions (models 1, 2, and 4). This may be due to three reasons: (1) predictive power differs from goodness of fit, that is, a model may fit a particular data set well even though the predictive power the model provides is small; (2) even though the added interaction term explained the largest proportion of variability in models 6 and 8, the total variability explained in all models was not large; and (3) the set of lines was relatively diverse. However, including the interaction terms G×E plus the additive × additive epistatic terms [main ($g_{Aj})$ and interaction term ($g_{A}E_{ij})]$ did not improve predictions by much, and the inclusion of these terms did not explain a large percentage of the total variability.

**Table 4:** Brier scores (mean, minimum and maximum; smaller indicates better prediction) evaluated for the validation samples

Model	Colombia			Zimbabwe			Mexico		
	Mean	Min	Max	Mean	Min	Max	Mean	Min	Max
1	0.3924	0.3798	0.4115	0.3617	0.3554	0.3698	0.3507	0.3386	0.3604
2	0.3869	0.3744	0.4011	0.3611	0.3542	0.3663	0.3434	0.3331	0.3572
3	0.3845	0.3733	0.4021	0.3628	0.3559	0.3701	0.3433	0.3302	0.3591
4	0.3856	0.3706	0.4024	0.3621	0.3538	0.3697	0.3431	0.3337	0.3526
5	0.3860	0.3734	0.4012	0.3619	0.3528	0.3734	0.3448	0.3251	0.3598
6	0.3261	0.3121	0.3402	0.3337	0.3249	0.3413	0.3145	0.2972	0.3295
7	0.3315	0.3170	0.3427	0.3308	0.3214	0.3363	0.3183	0.3003	0.3364
8	0.3249	0.3141	0.3417	0.3345	0.3247	0.3441	0.3189	0.3094	0.3277
9	0.3274	0.3159	0.3401	0.3327	0.3155	0.3455	0.3152	0.2981	0.3280

## Discussion

GS offers important opportunities to improve genetic gains in plant and animal breeding programs; however, more powerful estimation and prediction methods are required to fully exploit the advantages of GS. Most GS methods assume a Gaussian response; however, many important traits in plant breeding, such as disease resistance, percent protein content, and proportion of seed or plant damage, are not normally distributed and need special treatment. In this study, we extended the GBLUP method to ordinal categorical responses. To this end, we used the implementation available in BGLR with a probit link that can accommodate both binary and ordinal traits (Pérez-Rodríguez and de los Campos 2014). To our knowledge, this is the first study in GS that uses an ordinal threshold model to analyze a categorical response in plant breeding.

Most non-normal response variables are not linear with respect to model parameters and, unlike normal responses, the variance of the phenotypes is dependent on the mean. The GLMM uses: (1) a link function (typically nonlinear on the parameters) to specify the relationship between the mean of the response variable and a linear model; and (2) a variance function to describe the relationship between the mean and the variance of the distribution of the response variable, which enables it to relate traditional linear regression to non-normal data. This gives the GLMM framework a stronger basis for hypothesis testing and for more precise estimates of fixed and random effects. This statement applies to response variables coming from many different distributions, and not only to ordinal categorical responses.

GS methods have been implemented for threshold (ordinal categorical) traits in animal breeding. For example, González-Recio and Forni (2011) developed versions of BayesA, Bayesian Lasso, and two machine learning methods for dichotomous traits; Villanueva *et al.* (2011) also developed a version of BayesB for dichotomous traits; Wang *et al.* (2012) implemented methods BayesA, BayesB, and BayesCπ for ordinal categorical traits; and Kizilkaya *et al.* (2014) implemented BayesCπ for ordinal categorical traits. All these studies concluded that the traditional GS linear models are not suitable for threshold traits, since the basic assumptions of the linear model are violated, which produces a considerable reduction in prediction accuracy. However, to our knowledge, no plant breeding study so far has addressed the analysis of categorical traits. Our study fills this gap by providing a full description of a multithreshold GBLUP model (TGBLUP) and an empirical evaluation based on real data.

Prediction accuracy in GS usually is assessed using the sample correlation between predictions and phenotypes. However, this metric is not appropriate for categorical outcomes. For this reason, we used the Brier score for assessing the prediction ability of the proposed threshold model, which assigns a numerical score based on the predictive distribution. This scoring rule is “strictly proper” in the sense that it maximizes the expected score of an observation (and is unique) and allows making fair comparisons among models. To our knowledge, our study is the first one in GS to use this score for assessing prediction accuracy.

We presented nine specifications of the TGBLUP model which differ on the type of effects included. We considered main effects models and models for interactions between genetic factors (*e.g.*, additive ×additive epistatic) and between genetic and environmental factors *e.g.*, additive ×additive× environment interaction. For specifying these interactions, we used the framework proposed by Jarquín *et al.* (2014) within a threshold model framework. We found that models that take into account interactions (models 6−9) explained almost two times more variability than those without interaction (models 1−5). However, the interaction that was clearly most important was the G×E term; this effect captured the largest proportion of the total variability explained by the models (51.68% in model 6 and 50.19% in model 9), and was the one that led to the largest gain in prediction accuracy. This result is in agreement with previous studies that have highlighted the importance of modeling G×E in GS in plant breeding (Burgueño *et al.* 2012; Jarquin *et al.* 2014).

Inclusion of additive × additive epistatic terms did not explain much of the total variability, and did not help much to improve prediction ability. Also, we found that estimated threshold parameters could be clustered into two groups, one for those without interactions (models 1−5), and another for those with interaction terms (models 6−9). This was because including interactions increases the variance of the liability score and, therefore, changes in threshold values are needed to accommodate the observed probabilities of each of the categories.

Finally, although the analyses presented here used Gaussian priors for marker effects, the multi-threshold model used in this study can be implemented with any of the priors commonly used in GS, including those that induce differential shrinkage of estimates of effects or a combination of variable selection and shrinkage. The current implementation of BGLR allows users to do this.

We extended the GBLUP, a model commonly used in GS for analyses of normal traits, to situations where the response is ordinal (TGBLUP). We provided a detailed description of the model and introduced a metric, the Brier score, for assessing prediction accuracy of ordinal categorical outcomes. We presented an empirical evaluation using a (real) data set of 278 maize lines genotyped with 46,347 SNPs and evaluated for disease resistance using an ordinal categorical scoring system in three environments (Colombia, Zimbabwe, and Mexico). A total of nine models were used. In addition, we provide details of the R code used to implement these models using the BGLR package.

Our results highlight the importance of including G×E (capturing at least 49.42% of the total variability); when this interaction was taken into account, this increased the total variability explained by these models and increased prediction accuracy between 8 and 19% relative to models based on main effects only. Considering additive × additive epistasis did not produce a sizable increase in prediction accuracy.

## Appendix 1

### FITTING MODEL 9 TO RANDOMLY SAMPLED TRAINING AND TESTING SETS

library(BGLR)

library(matrixcalc)

# X, data frame containing the elements described below;

# - X$loc: (n×1), a factor giving the IDs for the environments (location);

# - X$line: (n×1), a factor giving the IDs for the varieties;

# - X$y: (n×1), a numeric vector for rating the 5 levels of the disease;

# - M: a matrix containing the genetic information (dimensions equal to the number of lines by the

# - number of SNPs [46,347]).

### Note: To replicate the results presented in this article, the sampling of training-testing sets must be done #according to the methods described in the article (70% training and 30% testing); this example simply #illustrates how to fit model 9 for a randomly chosen testing data set. The code for the other model is similar.

#Calculating the marker-derived genomic relationship matrix (GRM)

M<-scale(M,center=TRUE,scale=TRUE)

G<-tcrossprod(M)/ncol(M)

# Incidence matrix and covariance for main effects of environments.

ZE<-model.matrix(∼factor(X$loc)-1)

KE=tcrossprod(ZE);

# Incidence matrix and covariance for main effects of lines.

X$lines<-factor(x=X$lines, levels=rownames(M), ordered=TRUE)

ZL<-model.matrix(∼X$lines-1)

KL=tcrossprod(ZL);

# Genetic covariance structure of genetic effects in the full data

KG= ZL%*%G%*%t(ZL);

# Epistasis additive × additive covariance structure in the full data

GA=hadamard.prod(G,G)

KGG= ZL%*%GA%*%t(ZL);

# G×E covariance structure for the full data set

KGE=hadamard.prod(KG, KE)

diag(KGE)=diag(KGE)

KGE=KGE/mean(diag(KGE))

# GG×E Epistasis additive × additive structure for the full data set

KGGE=hadamard.prod(KGG, KE)

diag(KGGE)=diag(KGGE)

KGGE=KGGE/mean(diag(KGGE))

yNA<- X$y; n=length(X$y); p=round(0.30*n);

tst<-sample(1:nrow(X), size=p, replace=FALSE)

yNA[tst]<-NA

ETA<-list(ENV=list(K=ZE, model=’FIXED’), LINE=list(K=KL, model=’RKHS’),

G=list(K=KG, model=’RKHS’), GG=list(K=KGG, model=’RKHS’),

GE=list(K=KGE, model=’RKHS’), GGE=list(K=KGGE, model=’RKHS’))

fm9<-BGLR(y=yNA,response_type=’ordinal’, saveAt=’M9_’, nIter=52000, burnIn=6000)
